# Red Blood Cell Stiffness and Adhesion Are Species-Specific Properties Strongly Affected by Temperature and Medium Changes in Single Cell Force Spectroscopy

**DOI:** 10.3390/molecules26092771

**Published:** 2021-05-08

**Authors:** Dina Baier, Torsten Müller, Thomas Mohr, Ursula Windberger

**Affiliations:** 1 Decentralized Biomedical Facilities, Center for Biomedical Research, Medical University Vienna, 1090 Vienna, Austria; dina.baier@univie.ac.at; 2 Institute of Inorganic Chemistry, University of Vienna, 1090 Vienna, Austria; 3 Institute of Cancer Research and Comprehensive Cancer Center, Medical University Vienna, 1090 Vienna, Austria; thomas.mohr@mohrkeg.co.at; 4 JPK BioAFM, Bruker Nano GmbH, 12489 Berlin, Germany; to.mueller@bruker.com; 5 ScienceConsult–DI Thomas Mohr KG, 2353 Guntramsdorf, Austria

**Keywords:** atomic force microscopy, Young’s modulus, red blood cell, adhesion, deformability, animal species, comparative

## Abstract

Besides human red blood cells (RBC), a standard model used in AFM-single cell force spectroscopy (SCFS), little is known about apparent Young’s modulus (Ea) or adhesion of animal RBCs displaying distinct cellular features. To close this knowledge gap, we probed chicken, horse, camel, and human fetal RBCs and compared data with human adults serving as a repository for future studies. Additionally, we assessed how measurements are affected under physiological conditions (species-specific temperature in autologous plasma vs. 25 °C in aqueous NaCl solution). In all RBC types, Ea decreased with increasing temperature irrespective of the suspension medium. In mammalian RBCs, adhesion increased with elevated temperatures and scaled with reported membrane sialic acid concentrations. In chicken only adhesion decreased with higher temperature, which we attribute to the lower AE-1 concentration allowing more membrane undulations. Ea decreased further in plasma at every test temperature, and adhesion was completely abolished, pointing to functional cell enlargement by adsorption of plasma components. This halo elevated RBC size by several hundreds of nanometers, blunted the thermal input, and will affect the coupling of RBCs with the flowing plasma. The study evidences the presence of a RBC surface layer and discusses the tremendous effects when RBCs are probed at physiological conditions.

## 1. Introduction

Red blood cells (RBC) are the most common type of blood cells in vertebrates, and carry oxygen throughout the body. RBCs are required to be highly flexible in order to be able to pass through narrow capillaries and small vessels with diameters of less than 5 µm. This is accomplished by a homogenous, organelle-free cell interior surrounded by a composite membrane that supports all the equilibrium forces responsible for deformation [1]. Therefore, and, because of its unpretentious geometry, the human RBC is used as convenient model system to study membrane elasticity. The high surface-to-volume ratio of human RBCs facilitates the diffusion of oxygen, as well as proper cell folding without the need to macroscopically stretch out the submembrane’s filamentous cytoskeleton [2]. Elastic stretching is accomplished by the semiflexible spectrin–actin complex intermeshed with junctional protein complexes, being overlaid by an asymmetric phospholipid bilayer that is anchored to the cytoskeleton by several transmembrane proteins [3]. Besides human RBC’s mechanical properties, extensively studied under various conditions, little is known about the cellular characteristics elasticity and adhesion of phenotypically different RBCs/animal RBCs.

To gain a comprehensive view, the biomechanical properties of elasticity i.e., Young’s modulus (Ea), and adhesion were measured on selected RBC types that differ in shape, membrane composition, surface charge, and intracellular features (see Figures S1a and S1b) with atomic force microscopy (AFM) in the single cell force spectroscopy (SCFS) mode. Species selection was based on the resting shape of RBCs (discocytes, ellipsoids) and on key features like aggregability, cell stiffness, or the presence of a nucleus (Table 1). AFM is a nanoanalytical instrument used in medical sciences that allows probing of biomechanical and physicochemical properties of biomedical samples [4,5,6]. For this study we chose AFM-SCFS as an appropriate technique as it gives information about the area compressibility as well as adhesiveness of cells to the probing device, the cantilever, in a single test run. At the same time, the surrounding conditions can be adjusted i.e., by varying the test temperature and the suspending medium. Viscoelastic properties of RBCs have been investigated by several single cell techniques (for reviews see [7] and [8]). In many studies the cell exterior is removed by washing steps [9,10] and RBCs are probed at ambient temperature [11,12,13,14]. Besides the influences that the several test methods exert on the cell response [15,16,17], the environmental test settings may also modify the test outcomes. To assess the influence of altered test conditions on Ea and adhesion of the distinct RBC types, measurements were performed at 25 °C, 32 °C, and the respective body temperature (37 °C, except for chicken at 42 °C). Measurements at the intermediate temperature of 32 °C were included to monitor the profile of the temperature effect on Ea and adhesion. Moreover, we attempted to assess the feasibility of SCFS under largely physiological conditions by suspending RBCs in autologous plasma.

In this study, several knowledge gaps are being addressed. Firstly, one aim was to investigate the biomechanical characteristics of certain animal RBCs used as representative models for distinct phenotypical features within one comparative approach. The second aim was to obtain these properties under largely physiological conditions (at body temperature in autologous plasma) and to compare the outcomes with values obtained at simplified conditions (at 25 °C in aqueous NaCl solution). We were interested in whether the trend by which biomechanical properties change is similar for all of our investigated RBC phenotypes or if distinct, species-specific conclusions have to be drawn. In conducting the study, it was found that replacing the crystalloid 0.9% aqueous NaCl solution with autologous plasma completely changed the biomechanical properties of all the erythrocytes tested. Our findings open up a new view of how erythrocytes are embedded in plasma.

## 2. Results

### 2.1. AFM Tests: Apparent Young’s Modulus (Ea) in 0.9% Aqueous NaCl Solution

Ea-values in 0.9% aqueous NaCl solution display the local deformability of the RBC that is surrounded only by monovalent ions. The ranking of Ea at room temperature (RT, 25 °C) was the following: horse RBCs yielded the highest median value followed by camel, human fetal RBCs from umbilical cord blood, and ultimately adult human RBCs (Table 2). As poultry RBCs are nucleated, the results depended on whether the central or the peripheral region was indented. Indentation in the cell center resulted in five- to tenfold higher values as compared to probing immediately next to the nucleus. Ea above the central nucleus is therefore significantly higher than at the periphery. With temperature increase, Ea-values decreased in every species with exception of the central region of the poultry RBCs, which remained constant throughout all temperature variation. The ranking of Ea changed at body temperature 37 °C/42 °C (mammals/chicken, peripheral region). At body temperature, camel RBCs displayed the highest Ea-values, followed by horse, chicken, and human (fetal and adult values were comparable) RBCs, respectively. The highest temperature dependency of Ea-values was found with horse and human fetal RBCs, followed by chicken, camel, and ultimately adult human RBCs. Importantly, the higher the temperature became, the smaller were the differences of Ea-values between the species.

### 2.2. AFM Tests: Apparent Young’s Modulus (Ea) in Autologous Plasma

Force–distance curves were obtained from horse, camel, human adult and fetal RBCs in autologous plasma to test if Ea-values change with the physiologic surrounding. Overall, plasma significantly affected Ea as compared to values obtained in 0.9% aqueous NaCl solution (Table 2). The median values of all species at 25 °C, 32 °C, and 37 °C were lowered by mean factors 4.2, 2.6, and 2.5 as compared to 0.9% aqueous NaCl solution, respectively. The ranking of Ea at 25 °C was the following: camel RBCs yielded the highest median Ea followed by human fetal, human adult and horse RBCs. This order stayed steady throughout the temperature increase. Note that horse RBCs showed the highest Ea-values in 0.9% aqueous NaCl solution but the lowest Ea-values in plasma. Ea-values of horse RBCs also showed the least temperature dependency in plasma, whereas it showed the highest temperature dependency in 0.9% aqueous NaCl solution. Thus, horse RBCs softened most in plasma and with higher temperatures. Similarly to the observations in 0.9% aqueous NaCl solution: the higher the temperature, the lower were the differences between the species.

Taken together, Ea of RBCs differed strongly among the tested species and were significantly influenced by the choice of temperature and medium in a species-specific manner. Whereas horse RBCs were especially prone to changes in medium and temperature, RBCs from human adults were least influenced by the differing conditions. All species presented virtually equal median Ea-values at their respective body temperature in plasma, indicative for physiologically optimal conditions for RBC elasticity that might be species-independent.

### 2.3. AFM Tests: Adhesion in 0.9% Aqueous NaCl Solution

The adhesion of RBCs suspended in 0.9% aqueous NaCl solution is the result of the detachment of the retracting cantilever from the cell surface. We provide here the detachment work as adhesion parameter. At 25 °C, adhesion was highest in camel, followed by horse, human fetus, chicken and adult human RBCs (Table 3). The temperature profile was not uniform among the vertebrate classes. Adhesion increased with temperature in the mammals, but decreased in chicken. For body temperature, the ranking of the detachment work therefore changed to: horses > camels > human fetuses > human adults > chicken erythrocytes. Thus, adhesion and its temperature-dependency are species-specific properties.

### 2.4. AFM Tests: Adhesion in Autologous Plasma

Adhesion was completely absent in all tested RBC phenotypes when autologous plasma was added (compare with Figure S2). Raising the temperature did not elicit any other response.

Taken together, tests in 0.9% aqueous NaCl solution show that RBC adhesion is a species-specific property. The ranking of adhesion paralleled the ranking of Ea at RT, pointing to the influence of contact area to the detachment work of the cantilever. Since in chicken RBCs the adhesion decreased together with the Ea value, no such ranking can be provided for tests at higher temperature. Autologous plasma completely shielded the RBC surface, as the detachment of the cantilever from the cell surface required no work at any temperature (25, 32, 37 °C).

## 3. Discussion

To the best of our knowledge, this study is the first one that uses AFM-SCFS to probe animal RBC types, combining also avian and specific mammalian RBCs within one comparative approach. Species selection was based on the resting shape of RBCs (discocytes, ellipsoids) and on key features such as high aggregability (horse), cell stiffness (camel), cell inflation (human fetus), or the presence of a nucleus (chicken) (Table 1). In this comparative approach, we show that Ea and adhesion are species-specific properties depending on the individual RBC phenotype. These data (Table 2 and Table 3) may serve as a repository to study animal RBC behavior in flow. Moreover, animal RBCs offer specific characteristics that can be helpful in explaining pathophysiological conditions of human RBCs [39]. Hence, the obtained data of the different RBCs might be used as models for human RBCs under pathological conditions experiencing phenotypical changes. Regarding the search for a consistent trend in RBC behavior, we show that Ea and adhesion are similarly influenced by temperature and the presence of autologous plasma molecules. We are therefore including a technical note: not only is it important to use the same conditions when comparing study groups, but it must also be questioned whether values obtained under conditions that simplify the test procedures (ambient temperature, buffer medium) reflect and can mimic the circumstances in a physiological environment.

In detail, when we indented cells in NaCl solution, Ea decreased with increasing temperature regardless of size and shape of the cells throughout all tested RBC phenotypes, but the temperature profile was species-specific. As an effect of a higher cytoplasmic volume [40], inflated cells like fetal RBCs, display a higher Ea, as compared to adult human RBCs. In chicken, the region above the central nucleus did not respond to temperature changes and remained at almost constant stiffness, which can aid in limb perfusion when the blood temperature drops in wintertime due to the countercurrent blood flow [41]. Even though avian RBCs contain organelles and membranes are fortified by a tubular network and connected with the nuclear envelope by several intermediate fibers [37,40,42], Ea’s of chicken and human RBCs became comparable at higher temperature. This shows that the presence of intracellular elements does not automatically predict a high Ea when structures can move sideways. Such a dissipative property of the cytoplasm explains also the flow behavior of chicken RBCs in a network (Figure S3).

Adhesion increased in all mammalian RBC types with the rise of temperature when cells were probed in NaCl solution. This can be attributed to a larger contact area since Ea decreased in parallel [43]. Concerning hydrophilicity as another cause for species-specificity, we found congruence between the measured detachment work at RT and the reported sialic acid concentration of the tested species [24,25,28,29,32]. Both parameters show the same ranking: horse/human fetus/camel > human adult > chicken (due to the different methods used in the reports, the sialic acid concentration from horse, human fetus, and camel RBC cannot be further discriminated). These findings are additionally supported by previous reports showing a link between cellular adhesion and surface sialic acid concentration [44,45].

Only in the avian RBC type adhesion decreased with the rise of temperature even though, Ea decreased. Adhesion showed the lowest value in chicken at body temperature although the membrane has no difficulty in deforming to generate a good contact area (Figure S3, see also the temperature profile of chicken Ea-values in Table 2). Since a lowering of the contact surface can be excluded as the cause, the origin of this finding must be sought in other properties. The stabilization of the bilayer through integral proteins that provide also the anchor points to the cytoskeleton (e.g., band 3 protein, AE-1) must have an effect on adhesion. If a membrane is equipped with a high concentration of integral proteins, the bilayer area in between is reduced. This not only protects against tether extraction and membrane fragmentation as in camel RBCs, but also hinders thermal membrane undulations in order to reduce the steric repulsion between the erythrocyte surface and the cantilever [46] so that a higher adhesion can be achieved. In contrast to camel membranes that have a high AE-1 concentration [47], chicken membranes possess much fewer band 3 protein copies, and band 3 lacks the adherence to glyceraldehyde-3-phosphate dehydrogenase [36]. The decrease in adhesion in chicken RBCs, as the thermal stimulus got higher, might be associated with higher dynamic surface roughness due to a larger bilayer area in between the anchor points to the cytoskeleton.

When RBCs were indented in autologous plasma, the force-distance curves flattened and adhesion was completely abolished in every cell. As a technical note, we add that working in plasma can passivate cantilevers, so that no adhesion is measureable anymore [48]. The most important finding when working in plasma however, relates to the decrease in Ea, which was greater here than with changes in temperature. Particularly, Ea of horse RBCs was unaffected by temperature changes when measured in plasma, which is in clear contrast to findings in NaCl solution as suspending medium. Two causes can be formulated for the cantilever to cover a longer distance until it reaches the force setpoint: a change of membrane mechanics, or a change of cell size.

The first option concerns an uptake of plasma components into those membrane structures that provide bending resistance with the aim to weaken their cohesion. One has to consider first that the intimate cell contact surface is not the bilayer but the glycocalix. The glycocalix layer is considered a matrix [49] and serves as molecular sieve. To elicit a cellular response often requires high stoichiometric concentrations of agents or its collapse [50]. Compounds can intercalate within its endings and bind to them [51], but there is currently no evidence that the glycocalix layer provides a bending modulus to the RBC. The next cell layer is the bilayer, which is a barrier for the flux of hydrated molecules. When albumin reaches the bilayer it binds there unspecifically through hydrophobic interaction [52]. Only in specific cells albumin is transported intracellularly by endocytosis [53] and transcytosis [51]. This speaks against an intercalation of albumin in the bilayer. The higher molecular weight of other plasma proteins will sterically hinder their intercalation. From a mechanical point of view, the bilayer contributes only little bending rigidity to the membrane. It mainly dissipates energy inputs into a transversal displacement of its elements. The next structure is at the same time the main structure responsible for cell deformation [2]. The spectrin filaments together with the several other proteins that form the branchpoints of the cytoskeletal meshwork are located underneath the bilayer and are not accessible for the direct approach of plasma compounds. The RBC cytoskeleton deforms generally passively. Only one complex is actively triggered [54]. ATP production in RBCs depends on the availability of glucose as substrate and on the enzymatic activity of the Embden–Meyerhof pathway. Upon phosphorylation, the actin-protein-4.1R complex loses its connection to the bilayer, which allows a higher strain for the RBC at equal stress [55]. The only remaining cause for a gain in RBC deformability is the availability of glucose when we added plasma. However, we only had our erythrocytes in 0.9% aqueous NaCl solution for a period of 10 min so that they could settle on poly-l-lysine on the Petri dish before we replaced it with plasma. Very obviously, the cells remained in their homeostasis.

The second option, namely cell enlargement taking place, implies that the cantilever does not start to deflect close to the real cell surface, but at a certain distance away. This option presumes that a soft surface layer has been added to enhance the functional cell size. The thickness of this layer can be estimated from the degree of softening by replacing NaCl solution by plasma in analogy to tests of the endothelial cell glycocalix layer [56]. In this regard, horse RBCs have the largest halo followed by human fetal RBCs, camel RBCs, and RBCs from human adults. Figure S2 shows that in horses the gain in distance was in the range of hundreds of nanometers. This layer stabilized the Ea when we increased the temperature, so that the cell plus its surface layer was at similar stiffness at any temperature. Hence, the halo also made the human RBC types less temperature sensitive. Solely by the addition of plasma, this effect was generated. We did not treat the RBCs chemically to modify the cell surface but used the cells as drawn. This temperature-dependency is in clear contrast to tests in NaCl solution. In addition, we could extract tethers out of all RBC types except from camel RBCs only in NaCl solution, whereas in plasma tether extraction became impossible. The reason for this is because we did not reach the bilayer anymore.

Plasma buffered the membrane modulus in such a way that the RBC elasticity became independent of the species. Such property can only be present if the halo forms a stabile (solidlike/elastic) layer around the bilayer. Such elasticity most obviously stems from nanoconfinement of liquid plasma within the fibrous network of the glycocalix domains. Confined liquids exhibit mechanical properties that are different from those of a bulk [57]. Even water is elastic if it is confined [58]. In the body, the surface layer might be sheared off when RBCs flow, or its thickness becomes balanced by the adsorption and desorption of macromolecules [59].

## 4. Materials and Methods

### 4.1. Sample and Medium Preparation

#### 4.1.1. Blood Samples

Blood samples of four different vertebrate species (human, *Homo sapiens*, *n* = 6; horse, *Equus caballus*, *n* = 6; camel, *Camelus bactrianus*, *n* = 4; and chicken, *Gallus domesticus*, *n* = 5) as well as human fetal blood (*n* = 5) were used in this study. Blood was withdrawn immediately before the experiments (except for camel blood, which had a delay of three hours due to transportation) into K_2_-EDTA tubes (Greiner Bio-One GmbH, Kremsmünster, Austria) from the median cubital vein in the human volunteers (EK Nr.: 2114/2019), from the jugular vein in horses and camels (GZ: 66.009/0230-WF/V/3b/2016), from the wing vein in poultry (GZ: 68.205/0189-WF/V/3b/2015), and by puncturing the umbilical vein after removing the placenta following caesarean section (EK Nr.: 1602/2018).

#### 4.1.2. Sample Preparation

Autologous plasma was separated by centrifugation of human adult, human fetus, camel, and equine blood samples with avoidance of hemolysis. Vacutainers were centrifuged at 2000 rpm for 10 min at 25 °C. The supernatants were transferred into cryotubes and centrifuged at 3000 rpm at 25 °C to obtain platelet-depleted plasma devoid of any cell debris as far as possible. After the second centrifugation step, 2 mL of the supernatant were lifted off and used for measurements. One drop of freshly drawn blood was suspended in 1 mL of 0.9% aqueous NaCl (Fresenius Kabi Austria GmbH, Austria) to ensure a concentration suitable to perform single cell measurements. All measurements were performed on solid support cell culture dishes. For force spectroscopy, the RBCs were immobilized on the cell culture dish surface. Therefore, a designated area in the center of the dish was coated with 50 µL 0.005% poly-l-lysine (Sigma-Aldrich, St. Louis, MO, USA) and incubated for 10 min at room temperature. After incubation, the excess poly-l-lysine was removed. 50 mL of the RBC dispersion were distributed upon the poly-l-lysine coated area and incubated for 10 min. Finally, the samples were brought to a total volume with either 2 mL of 0.9% aqueous NaCl solution or, where possible, meaning without limitation by a low sample volume (all five chicken, three of the four camel samples), autologous plasma. If the measurements were performed at a temperature higher than 25 °C (RT), the samples were tempered for at least 10 min. The samples were brought to temperature using a dish heater (JPK Instruments AG, Germany). For measurements in autologous plasma, the tests were started after a period of at least 20 min for settlement of colloids.

### 4.2. AFM Tests

#### 4.2.1. AFM Setup

For force spectroscopy measurements studying RBC elasticity and adhesion, the CellHesion^®^ methodology (CellHesion^®^ 200, JPK Instruments AG, Germany) was used. The setup was placed on a Zeiss Microscope Axio Observer.A1 (Carl Zeiss Microscopy, Germany) and equipped with a temperature control unit (JPK Instruments AG, Germany). For the experiments the extend z-piezo with 100 µm of the CellHesion^®^ 200 system was used. The cantilevers employed in this study were made of low stress silicon nitride (Hydra-All, AppNano, Applied NanoStructures, Inc., CA, USA) with a nominal spring constant of 0.085 N/m, triangular shape and a back-side gold coating enhancing the laser reflection. The tetrahedral tip was made of uncoated silicon with a radius of curvature < 8 nm.

#### 4.2.2. Force Measurements

Prior to the measurements the sensitivity of the cantilever and the spring constant were evaluated in order to prevent artifacts associated with contamination of the tip from affecting the measurement. The cantilever sensitivity was determined from the force-distance curves recorded in clean cell culture dishes containing saline. The spring constant was determined by thermal noise analysis [60]. The single cell force spectroscopy was carried out in contact mode and operated with constant force up to 1 nN. The force–distance curves were recorded individually without contact time at 25 °C, 32 °C and the species’ respective native body temperature in saline and autologous plasma. The extend and retract speed was constantly 5 µm/s. The local mechanical probing was controlled by observing every measurement through a CCD camera (DFK31AF03, The Imaging Source Europe GmbH, Germany) mounted onto a Zeiss Microscope camera (bright field microscopy, Carl Zeiss Microscopy, LLC, NY, USA). Hence, it could be visually verified that the RBCs were indented at the inner area. Before the approach, the uncoated tip was lowered proximate on to the RBC. The tip indented the RBC membrane without penetrating it. The apparent Young’s modulus (Ea) was calculated from the contact region (green line, see Figure S2) of the force–distance curve by employing the Hertz–Sneddon model [61] of the data analysis software (CellHesion^®^ 200, version 4.2, JPK Instruments AG, Germany). The green line also shows our approach to calculate Ea. We indented cells up to 1 micron, which is more than 10% of the cell height, but calculated Ea at lower indentation depth to avoid the influence of the substrate (Petri dish) on Ea values. This approach is similar to the calculation of the zero force contact image, where the force-dependent topography and nanomechanic was calculated from raw data [62]. After reaching the maximum force of 1 nN, the cantilever was retracted from the RBC surface, which was eventually accompanied by adhesion of the cell membrane to the cantilever tip. The adhesion was observed as detachment work in the withdrawal region of the retract curve. It was determined quantitatively by acquisition of the area under the curve (AUC) [63]. Within each probe, at least ten RBCs were indented on average seven times. Consequently, each scanning cycle consisted of at least 70 data curves at each temperature and for each sample in the respective medium. The measurements were carried out on different RBCs ensuring physiological variability of the cells. Membrane rupture was not observed during indentation in none of the RBCs. In one human sample of our cohort, ten designated RBCs were measured at different temperatures in NaCl solution to confirm the decrease in stiffness with increasing temperature on the single cell level elucidating that it is not a cohort effect but also applies for a respective single cell (Figure S4).

#### 4.2.3. Theoretical Model and Data Processing

At the contact point of the extend phase the elastic deformation of the sample can be related to its Young’s modulus. To link the measured quantities to the Young’s modulus it is necessary to consider the sample deformation i.e., indentation δ. Before data processing, a crucial step in order to determine δ is the subtraction of the cantilever bending x from the height signal z. Tip-sample separation is the corresponding function in the used data processing software. The application of force by the cantilever on the RBC generates elastic deformation of the cell. Elastic deformation is described by several theories [64,65,66,67]. These theories are only approximations and, thereby, not entirely valid for a biological cell. Though, the Hertz–Sneddon model has proven to be a useful method to quantify elastic properties of materials [61]. The adaption of the model to the case of a tetrahedral tip shape was respectively given by Bilodeau [68].

In this regard, however, since some characteristics of the classical Hertz–Sneddon model are not fulfilled in biological samples, such as amongst others (i) ideal elasticity, (ii) homogenous material, (iii) no direct influence of the sample by the probe, the Young’s Modulus will herein be referred to as apparent Young’s modulus or RBC stiffness/elasticity.

### 4.3. Statistics

**Scheme 1:**
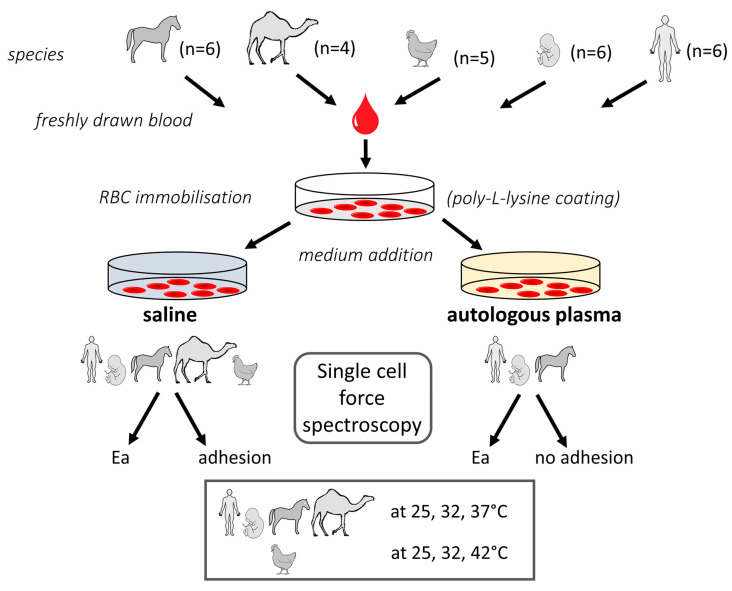
Representation of the experimental setting used in the present study. If graphical building blocks were not created, they were provided by Motifolio (license holder: Thomas Mohr).

A total of 1290 RBCs was indented (360 human RBCs; 240 human fetal RBCs; 300 horse RBCs; 240 camel RBCs; 150 chicken RBCs), including all test temperatures and both media (Scheme 1). Each of these 1290 cells was indented seven times on average to generate apparent Young’s modulus and adhesion for each cell. The number of force-distance curves that were evaluated is given in Table 2 and Table 3. Statistical analyses were carried out using GraphPad Prism 5.0 software (GraphPad Software, Inc., La Jolla, CA, USA). Statistical significance of differences was tested using one-way ANOVA followed by Tukey’s multiple comparison test. A *p*-value < 0.05 was considered statistically significant.

## 5. Conclusions

This study demonstrates that the RBC biomechanical parameters of apparent Young’s modulus (Ea) and adhesion vary strongly among different vertebrate species, presenting individual RBC characteristics (Table 1). Ea and adhesion can be considered as species-specific parameters depending on the RBC phenotype. Hence, the different RBC phenotypes might be used as models for human RBCs under pathological conditions experiencing morphological changes. The effect of thermal input and of autologous plasma on Ea can be considered as valid for RBCs in general with exceptions and gradual differences existing in form of species-specificity. The assessed data on Ea and adhesion of these RBC phenotypes in a direct comparative approach may serve as an important repository for future experiments, attributing also the change of parameters under largely physiological as compared to simplified test conditions. Most single cell approaches probe on closed systems in an artificially created environment as single cells would never be present as such. This lack of their natural connective system deprives many complex and dynamic interactions that would only be present under physiologic conditions. This study clearly demonstrates the dramatic effect of altered test conditions on RBC behavior. Our data also point to the presence of an elastic halo around RBCs that reduces or even abolishes the thermal input when the RBCs are surrounded by plasma. This will affect the coupling between RBCs and the flowing plasma and will subsequently influence the flow of RBCs in the body.

## Figures and Tables

**Table 1:** Phenotypical characteristics of selected RBC types tested in this study.

RBC Type	RBC Shape and Size	Aggregation Index M1	Elongation Index EImax	Membrane Proteins (Differences to Human)	Sialic Acid Content (Differences to Human)	Intracellular Structures
Human adult 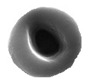	biconcave diameter 7.5–8.7 μm [18]	28.5 ± 6.6 [19]	0.618 (0.612/0.623) [19]	-	-	none
Human fetus 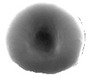	11% wider diameter than adult [20]	4.84 ± 0.89 [21]	higher rigidity index [21], lower shear elastic modulus [22]	comparable [23]	increased [24,25]	none, 21% bigger cell volume [20]
Horse 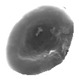	biconcave diameter 5.7 µm	55.9 ± 13.4 [19]	0.679 (0.590/0.878) [19]	deficient in band 4.2 and band 6 [26,27]	increased [28,29]	none
Camel 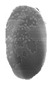	elliptic dimension 7 × 4 µm [30]	none [31]	n.a.	higher amount of band 3, less mobile [32,33]	increased [32]	none
Chicken 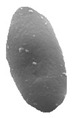	elliptic dimension 12.1 × 7.3 µm nucleus: 4.2 × 2.8 µm [30]	none	n.a.	less band 3 copies, no adherence to GAPDH [34,35,36]	lower [29]	nucleus, mitochondria, marginal band, filaments [37,38]

**Table 2:** Apparent Young’s modulus (Ea) of different vertebrate RBCs tested at 25, 32, and 37 or 42 °C in 0.9% NaCl solution or autologous plasma. * The 40 camel RBCs that were indented in autologous plasma stem from only one animal. In chicken, the value depended on the localization of indentation: ** Indentation above the nucleus (only median value provided). *p*-values in the Table indicate differences between Ea at the higher versus the lower temperature. For all species *p*-values were <0.001 when Ea in 0.9% aqueous NaCl solution was compared with the Ea in autologous plasma at the respective temperatures (e.g., at 25 °C in NaCl solution vs. 25 °C in plasma). Values obtained at the respective body temperature of the species are set in *italics*.

Apparent Young’s Modulus (Ea; in Pa):			Percentile				
Species	Medium	Temperature (°C)	50% (Median)	25%	75%	*p*-Value (Comparing Temperatures)	RBCs Tested/Force-distance Curves Evaluated
human adult	0.9% NaCl	25	251	199	332		60/397
		32	169	125	226	0.001	60/413
		*37*	*179*	136	238	*n.s.*	60/400
	autologous plasma	25	144	101	224		60/187
		32	126	91	162	*n.s.*	60/200
		*37*	*97*	*63*	*141*	*0.05*	*60*/184
human fetus	0.9% NaCl	25	754	527	954		50/357
		32	236	167	344	0.001	50/343
		*37*	*176*	*131*	*234*	*0.001*	*50*/341
	autologous plasma	25	217	138	299		30/122
		32	133	103	177	0.01	30/113
		*37*	*100*	*80*	*175*	*n.s.*	*30*/123
horse	0.9% NaCl	25	1260	729	1753		60/397
		32	524	346	788	0.001	60/422
		*37*	*351*	*185*	*742*	*n.s.*	*60*/406
	autologous plasma	25	126	92	183		40/200
		32	105	72	158	*n.s.*	40/160
		*37*	*96*	*71*	*138*	*n.s.*	*40*/181
camel	0.9% NaCl	25	825	565	1184		40/276
		32	489	320	697	0.001	40/296
		*37*	*439*	*345*	*600*	*n.s.*	*40*/260
	autologous plasma	25	583	272	863		40*/513
		32	203	140	309	0.001	40*/455
		*37*	*164*	*109*	*221*	*n.s.*	40*/463
chicken	0.9% NaCl	25	**2278/461	261	721		50/348
		32	**1914/282	155	445	0.001	50/302
		*42*	**2384/183	*91*	*427*	*0.01*	*50/274*

**Table 3:** Adhesion (detachment work; in aJ) of the different vertebrate RBCs tested at 25, 32, and 37 or 42 °C in 0.9% aqueous NaCl solution calculated from the AUC of the retract curve. Adhesion increases in mammalian RBC types when the temperature is raised in contrast to the avian RBC type. No adhesion was present when RBCs were suspended in autologous plasma. *p*-values in the Table indicate differences between adhesions at the higher versus the lower temperature. Values taken at the body temperature of the species are set in *italics*.

Adhesion (in aJ):			Percentiles				
Species	Medium	Temperature (°C)	50% (Median)	25%	75%	*p*-Value (Comparing Temperatures)	RBCs Tested/Force-Distance Curves Evaluated
human adult	0.9% NaCl	25	18	9	128		60/397
		32	31	15	63	0.001	60/413
		*37*	*44*	*21*	*149*	*0.05*	*60*/400
human fetus	0.9% NaCl	25	74	42	112		50/357
		32	108	57	168	0.001	50/343
		*37*	*137*	*89*	*221*	*0.01*	*50*/341
horse	0.9% NaCl	25	90	51	174		60/397
		32	68	42	175	*n.s.*	60/422
		*37*	*171*	*51*	*252*	*0.001*	*60*/406
camel	0.9% NaCl	25	143	55	194		40/276
		32	67	32	122	0.001	40/296
		*37*	*159*	*83*	*239*	*0.001*	*40/260*
chicken	0.9% NaCl	25	35	14	66		50/348
		32	32	2	97	0.01	50/302
		*42*	*7*	*1*	*23*	*0.001*	*50/274*

## Data Availability

Not available.

## Supplementary Materials

The following are available online, Figure S1: SEM images of RBC phenotypes and RBCs during indentation in bright field, Figure S2: force-distance curves of one identical RBC in saline and blood plasma, Figure S3: two poultry RBCs meet at a Y-junction in a PDMS microfluidic network. Figure S4: Ea of 10 human RBCs tested repeatedly at three temperatures in NaCl solution.

## Sample Availability

Not available.
