# Supplementary material for: Red Blood Cell Stiffness and Adhesion Are Species-Specific Properties Strongly Affected by Temperature and Medium Changes in Single Cell Force Spectroscopy
Source: Molecules. 2021 May 8;26(9):2771. doi: 10.3390/molecules26092771 (PMC8125892; doi:10.3390/molecules26092771)
Supplement: Supplementary file 1 [file molecules-26-02771-s001.zip › molecules-1133851-supplementary.pptx]

## Slide 1
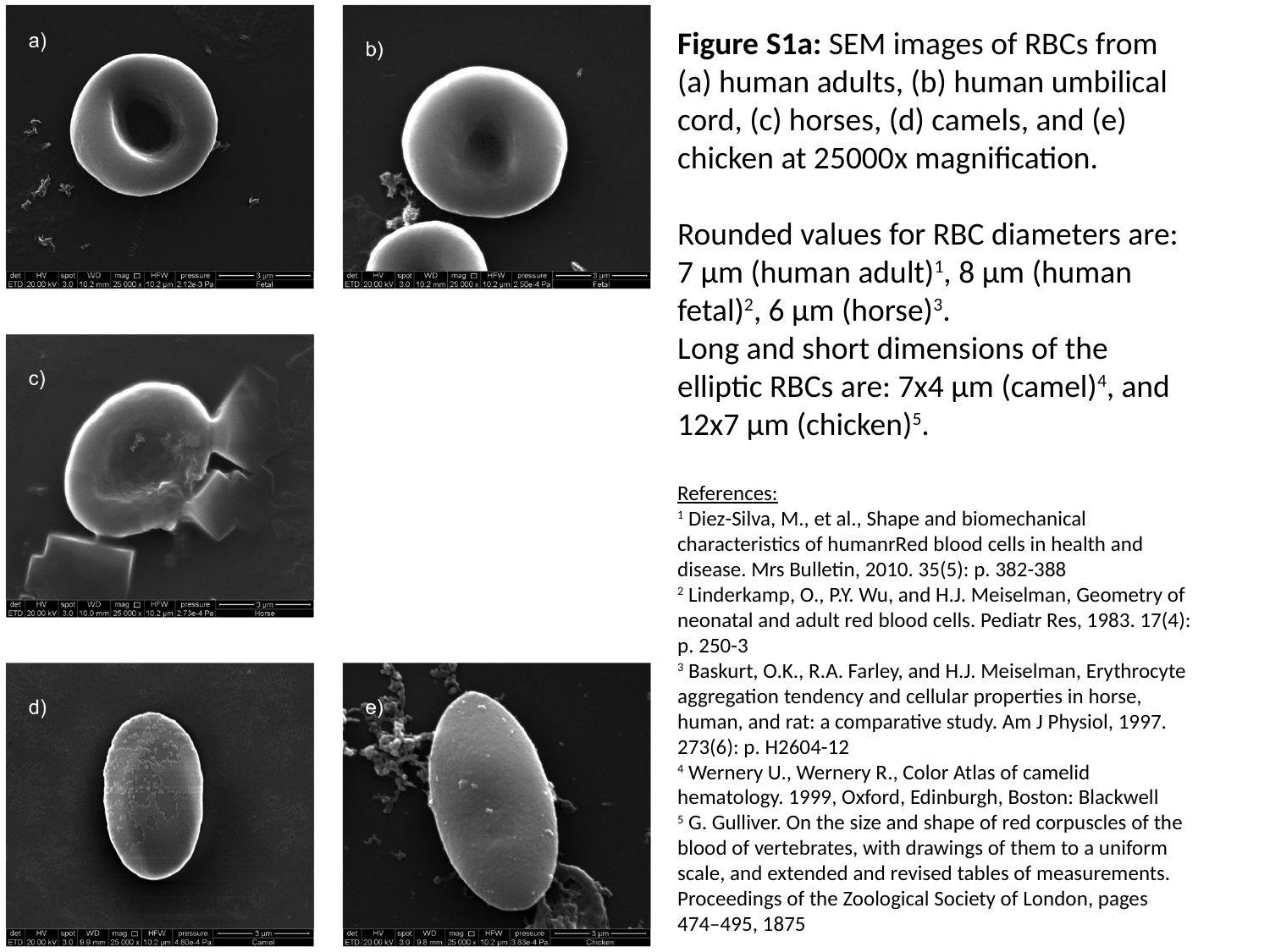

Figure S1a: SEM images of RBCs from (a) human adults, (b) human umbilical cord, (c) horses, (d) camels, and (e) chicken at 25000x magnification.
Rounded values for RBC diameters are: 7 μm (human adult)1, 8 μm (human fetal)2, 6 µm (horse)3.
Long and short dimensions of the elliptic RBCs are: 7x4 µm (camel)4, and 12x7 µm (chicken)5.
References:
1 Diez-Silva, M., et al., Shape and biomechanical characteristics of humanrRed blood cells in health and disease. Mrs Bulletin, 2010. 35(5): p. 382-388
2 Linderkamp, O., P.Y. Wu, and H.J. Meiselman, Geometry of neonatal and adult red blood cells. Pediatr Res, 1983. 17(4): p. 250-3
3 Baskurt, O.K., R.A. Farley, and H.J. Meiselman, Erythrocyte aggregation tendency and cellular properties in horse, human, and rat: a comparative study. Am J Physiol, 1997. 273(6): p. H2604-12
4 Wernery U., Wernery R., Color Atlas of camelid hematology. 1999, Oxford, Edinburgh, Boston: Blackwell
5 G. Gulliver. On the size and shape of red corpuscles of the blood of vertebrates, with drawings of them to a uniform scale, and extended and revised tables of measurements. Proceedings of the Zoological Society of London, pages 474–495, 1875

## Slide 2
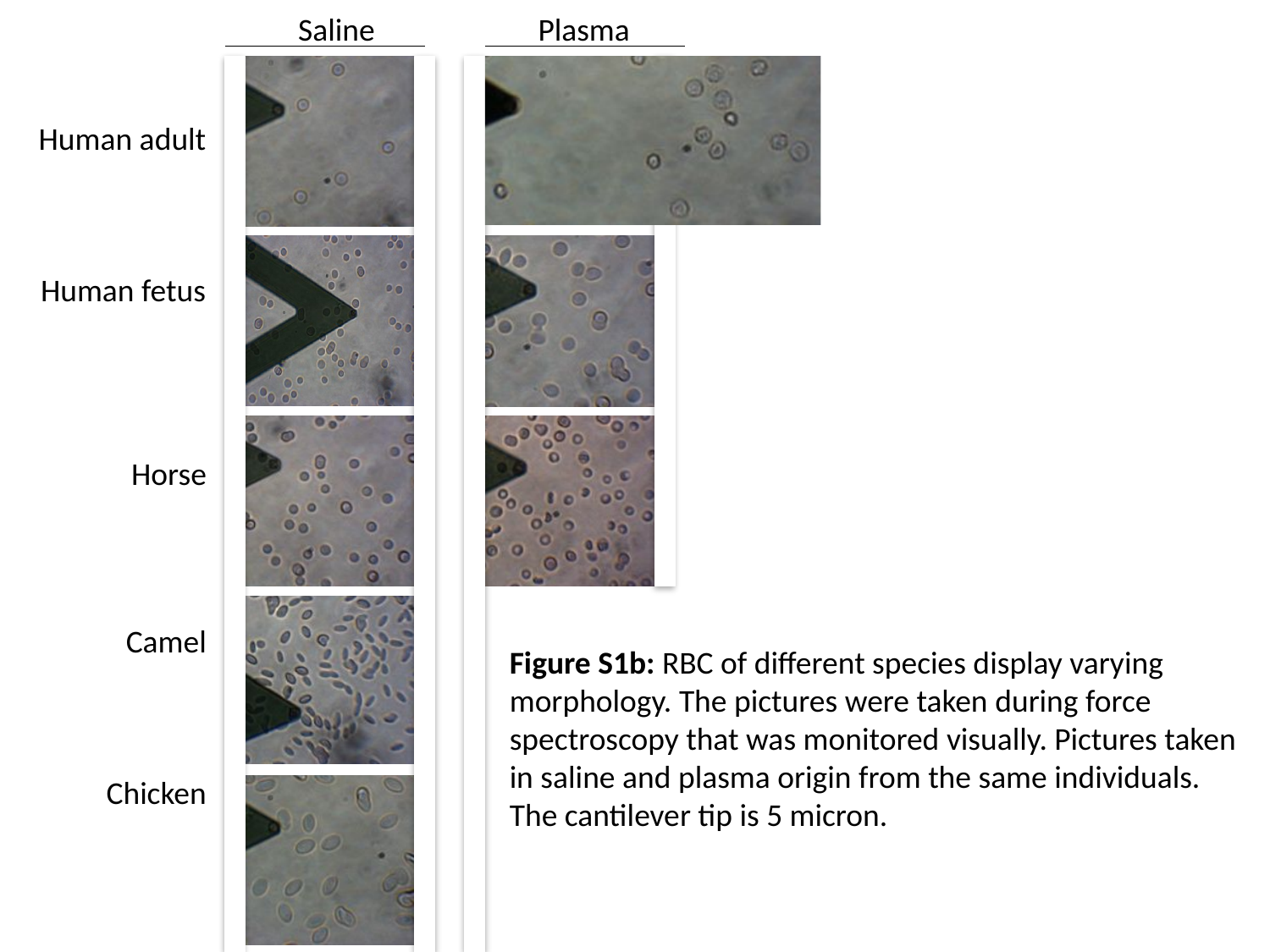

Saline
Plasma
Human adult
Human fetus
Horse
Camel
Figure S1b: RBC of different species display varying morphology. The pictures were taken during force spectroscopy that was monitored visually. Pictures taken in saline and plasma origin from the same individuals. The cantilever tip is 5 micron.
Chicken

## Slide 3
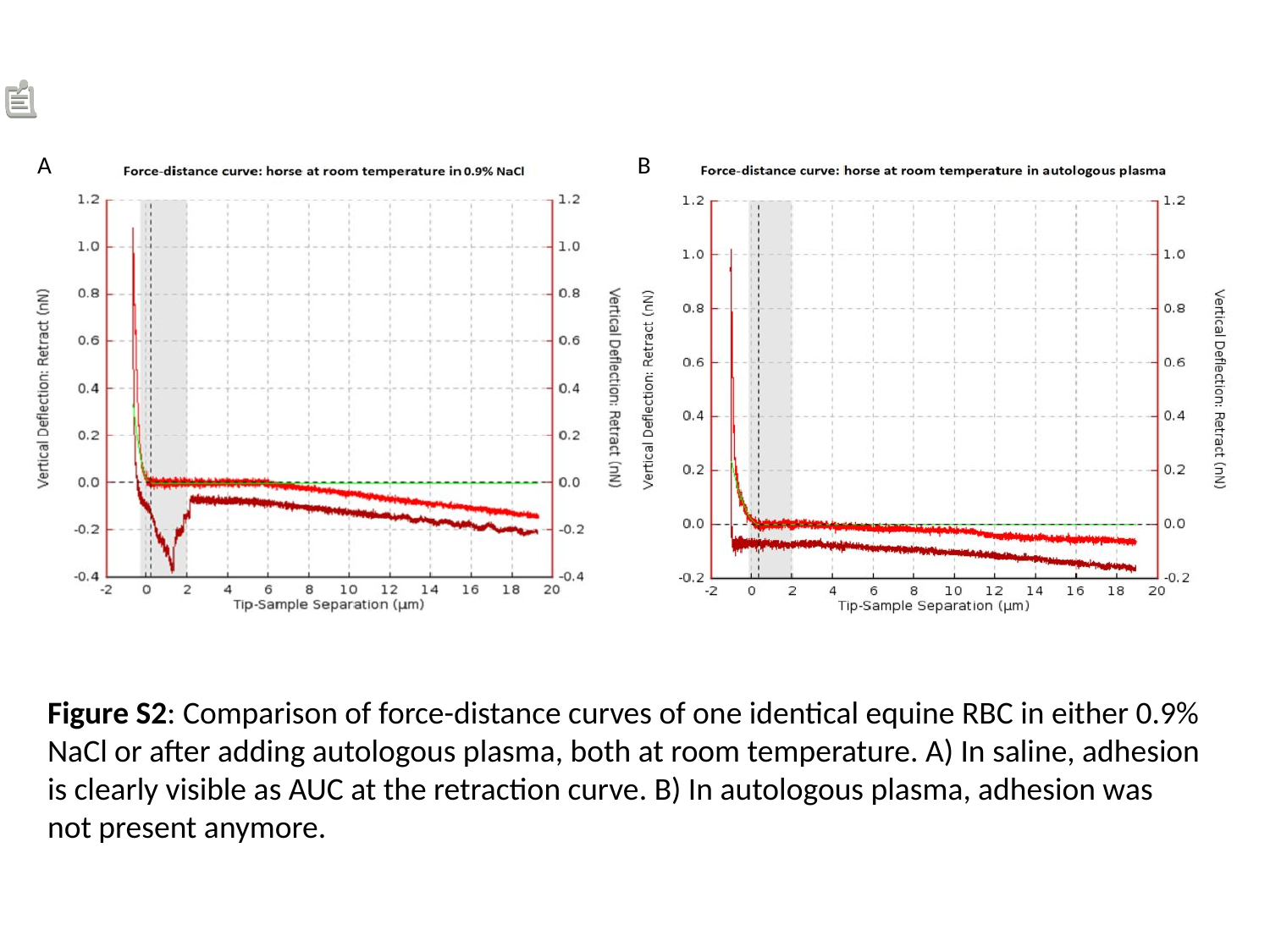

A
B
Figure S2: Comparison of force-distance curves of one identical equine RBC in either 0.9% NaCl or after adding autologous plasma, both at room temperature. A) In saline, adhesion is clearly visible as AUC at the retraction curve. B) In autologous plasma, adhesion was not present anymore.

## Slide 4
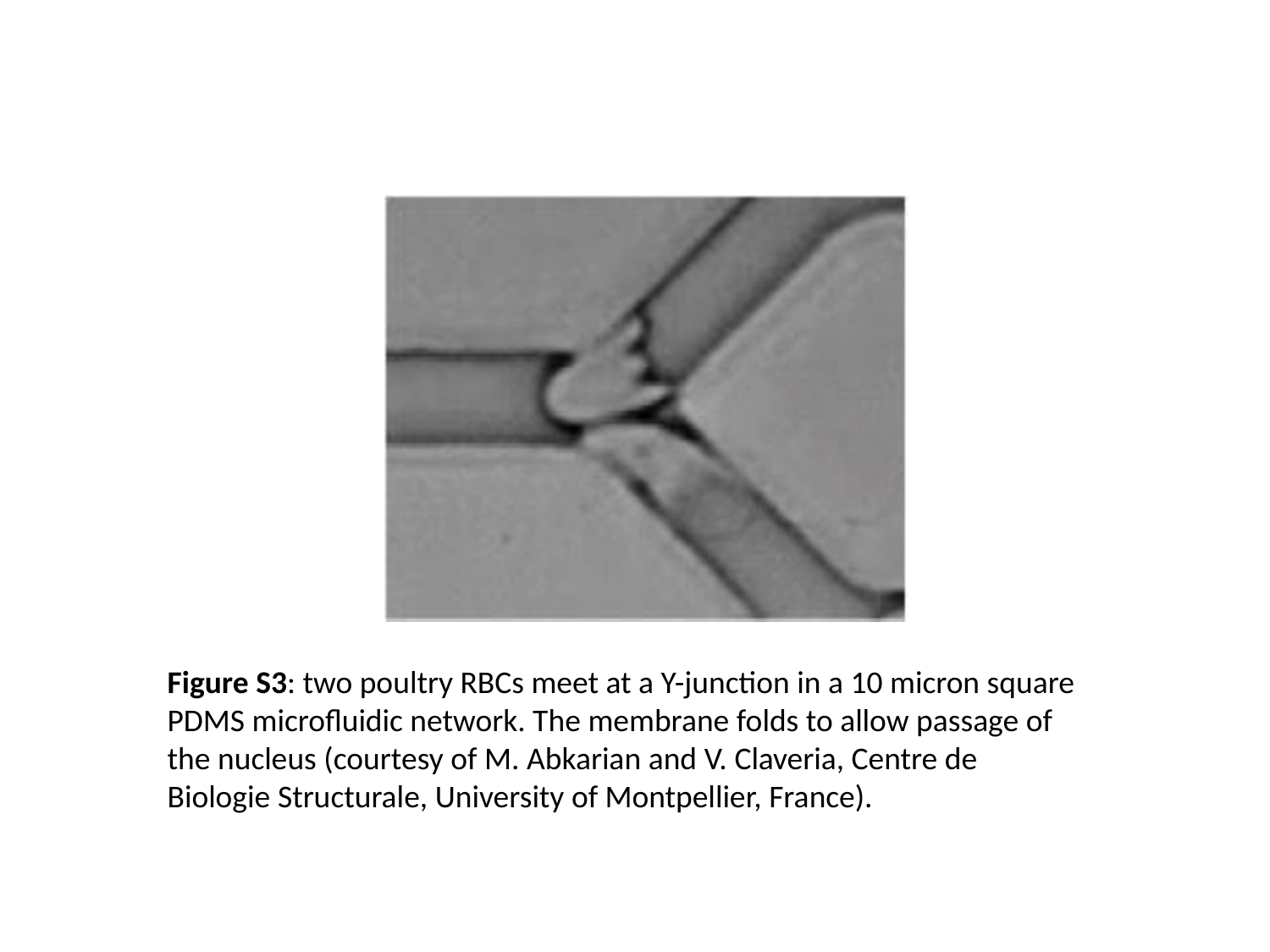

Figure S3: two poultry RBCs meet at a Y-junction in a 10 micron square PDMS microfluidic network. The membrane folds to allow passage of the nucleus (courtesy of M. Abkarian and V. Claveria, Centre de
Biologie Structurale, University of Montpellier, France).

## Slide 5
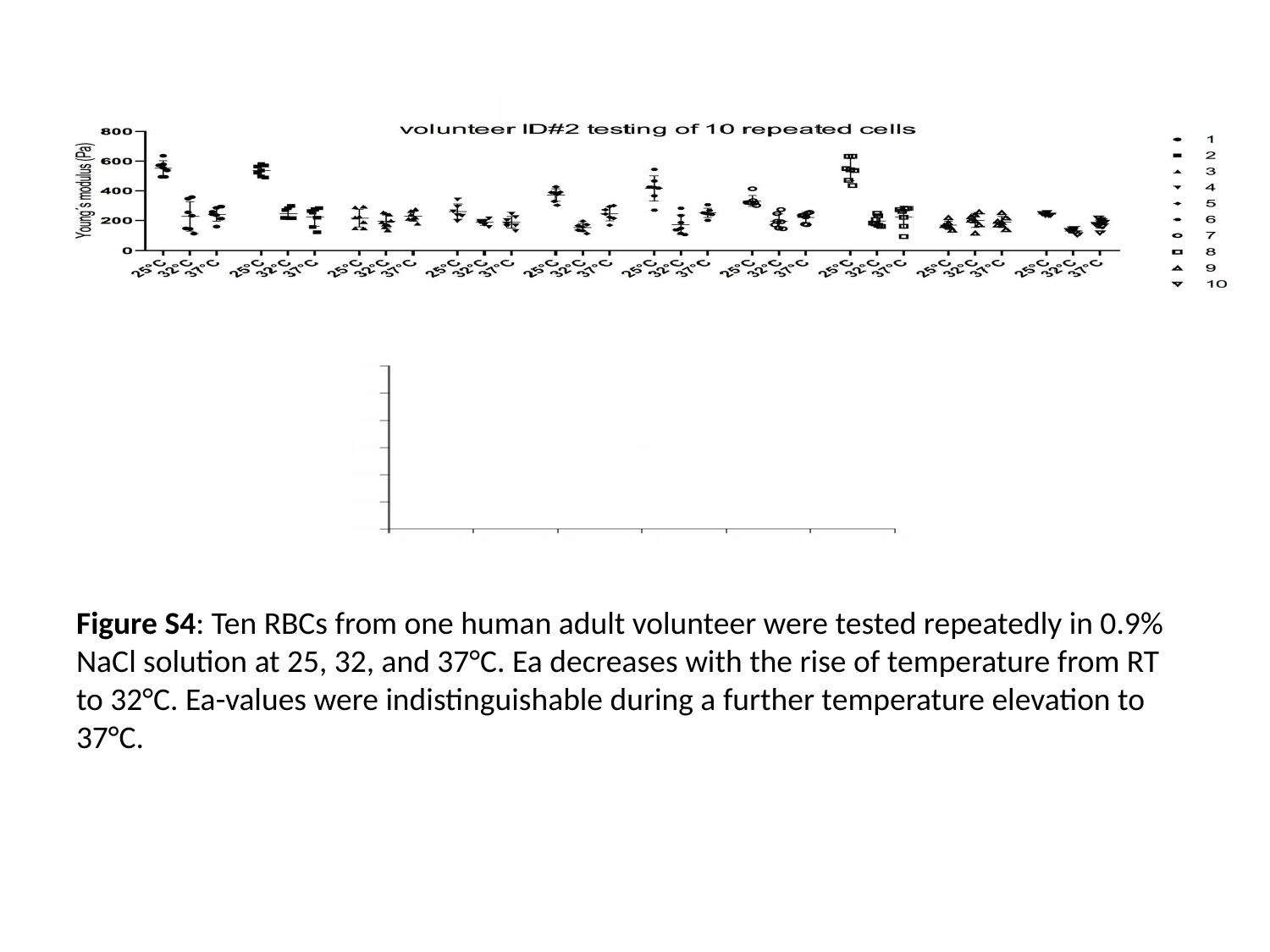

Figure S4: Ten RBCs from one human adult volunteer were tested repeatedly in 0.9% NaCl solution at 25, 32, and 37°C. Ea decreases with the rise of temperature from RT to 32°C. Ea-values were indistinguishable during a further temperature elevation to 37°C.
